# Burnout and posttraumatic stress symptoms in police officers exposed to traumatic events: the mediating role of ruminations

**DOI:** 10.1007/s00420-021-01689-9

**Published:** 2021-04-27

**Authors:** Nina Ogińska-Bulik, Zygfryd Juczyński

**Affiliations:** 1 Institute of Psychology, University of Lodz, Lodz, Poland; 2 Social Academy of Sciences, Institute of Applied Psychology, Lodz, Poland

**Keywords:** Traumatic events, Post-traumatic stress disorder, Burnout, Ruminations, Police officers

## Abstract

**Purpose:**

Police work carries the risk of burnout in the form of exhaustion and disengagement from work. Police officers are also exposed to traumatic events and the development of PTSD. The main aim of the cross-sectional study was to determine the mediating role played by rumination in the relationship between burnout and PTSD among police officers. It also examines whether burnout is a significant prognostic factor for PTSD symptoms.

**Methods:**

Data were obtained from a sample of 120 police officers. Of these one hundred, mostly men (83%), aged 23–47 years (*M* = 33.06, SD = 5.61), confirmed the experience of traumatic events in connection with their professional work. Three standard measuring tools were used: The Posttraumatic Checklist for DSM-5, The Oldenburg Burnout Inventory OBI, and The Event-Related Rumination Inventory.

**Results:**

The introduction of intrusive ruminating as an intermediary variable made the relationship between job burnout and PTSD non-significant, which indicates full mediation. The introduction of deliberate rumination as a mediator weakens the relationship between burnout and PTSD, which indicates partial mediation. It indicates that police officers who are burnout and who additionally tend to ruminate about experienced traumatic events are more likely to PTSD than police officers who are only burned out.

**Conclusion:**

Intervention programs for police officers should focus on strengthening stress management resources in the form of developing deliberate ruminations, thus allowing the experienced situations to be given a new meaning and to allow better coping.

## Introduction

As with all representatives of the emergency services, police officers are exposed to many stressors associated with their work. These concern tasks associated with everyday duties, such as providing assistance, mediating in conflicts between citizens, organizing road traffic or detecting crimes, as well as with traumatic events related to the risk of injury and death. The experience of the latter type of stressor leads to many negative consequences, among which posttraumatic disorder (PTSD) and burnout deserve special attention.

PTSD is characterized primarily by symptoms of intrusion, avoidance and hyperarousal. In the new classification of diseases (Diagnostic and Statistical Manual of Mental Disorders—DSM-5), this description has been supplemented by two more symptoms: negative changes in cognition and mood (APA 2013). These symptoms occur primarily in response to health- or life-threatening events and may be experienced directly or indirectly: in the latter case, the patient is a direct witness to how someone else experienced such an event, or how a traumatic event affected a close relative or friend.

American research indicates that between 12 and 35% of police officers suffer from PTSD; this value is much higher than the 1–2% observed in the case of the general population (Aamodt and Stalnaker 2006; Hartley et al. 2013). In turn, Canadian sources (Marchand et al. 2015) indicate that 6–35% of officers have been exposed to trauma in connection with the performance of their duty. Polish research shows that 57% of policemen experienced traumatic events related to their work, of which almost 50% of them had experienced it more than once (Dudek 2003). As well as in Poland (Basinska and Daderman 2014), high levels of burnout symptoms among police officers have been reported by studies in Spain (De la Fuente Solana et al. 2013), Sweden (Backteman-Erlanson et al. 2013), Turkey (Aydin and Tekiner 2016) and various other countries.

Police work is also associated with the appearance of burnout. Occupational burnout is a pathological syndrome that develops in response to long-term, chronic, interpersonal and occupational stress. It is defined as a prolonged response to stressors at work and includes symptoms of exhaustion, cynicism and inefficacy (Maslach et al. 2001). A new approach to this syndrome focuses on exhaustion and disengagement as the main dimensions of burnout (Demerouti et al. 2003, 2010). In this context, *exhaustion* refers to being physically, cognitively and emotionally drained of energy as a result of exposure to job demands while *disengagement* is expressed as distancing oneself from work and adopting a negative attitude toward work-related objects and tasks. General exhaustion, which refers to both withdrawing oneself from work and fostering negative attitudes toward one’s work, is related to the *energy* dimension, while disengagement, which is a broader construct than depersonalization, is related to the *identification* dimension.

The problem of occupational burnout has long been considered in various psychological studies but is now taking on special significance. In the new, 11th revision of the International Classification of Diseases (ICD-11), occupational burnout was formally recognized by the WHO as an official medical diagnosis. In employees exposed to traumatic events, burnout may increase susceptibility to PTSD, and may also precede the appearance of PTSD symptoms. The explanation for this relationship can be found in two developmental models of demand and resource imbalance: The *Job demand model* (JD-R) and the *Conservation of resources model* (COR). The JD-R model focuses on the concept of occupational burnout when individuals experience a constant demand for work, for which they have insufficient resources to meet and reduce them (Maslach and Leiter 2016). In turn, according to the COR, exposure to a wide range of stressors can deplete resources, resulting in emotional exhaustion and reduced motivation to perform professional tasks. The symptoms of burnout may increase the likelihood of specific negative consequences, including PTSD, after exposure to specific stressors such as traumatic events (Hobfoll and Freedy 1993). Professional burnout, as emphasized by Basińska and Gruszczyńska (2017), is associated with depletion of energy, which makes it a main factor in the health impairment process.

It is believed that occupational burnout may precede the development of further mental health disorders (Basinska and Gruszczyńska 2017; Wapperom 2016). However, few studies indicate a link between occupational burnout and PTSD, and no such studies have been performed for police officers. Positive relationships were found between PTSD and burnout symptoms in Greek (Katsavouni et al. 2016) and Korean firefighters (Jo et al. 2018). Burnout was also found to be an important prognostic factor for PTSD symptoms in crisis management specialists (LaFauci Schutt and Marotta 2011). Additionally, a cross-sectional study by Mealer et al. (2009) found that 98% of nurses surveyed who had met the PTSD diagnostic criteria were also positive for at least one of the three symptoms of burnout, as measured by the *Maslach Burnout Inventory*. Longitudinal studies conducted on military personnel deployed in Afghanistan confirmed that burnout is a predictor of PTSD (Wapperom 2016). Additionally, while job burnout was found to increase the risk of secondary traumatic stress disorder (STSD) symptom development, these symptoms were not related to job burnout at follow-ups in human service workers (Shoji et al. 2015).

In the cognitive model of trauma, special attention is paid to the negative assessment of the traumatic event experienced, mainly as a threat. Such appraisal generates not only negative emotions, but also persistent thinking (ruminating) about the experienced event, which contributes to the maintenance of PTSD (Ehlers and Clark 2000). Research indicates that individual cognitive activity in the face of trauma, especially in the form of ruminations, is one of the most important factors affecting the consequences of events, including PTSD (Ehlers and Clark 2000; Foa and Rothbaum 1998; Horowitz 1986; Janoff-Bulman 1992). Trauma-related rumination is characterized by repetitive thoughts about a negative, traumatic event and its consequences (Michael et al. 2007). This type of rumination is most often associated with pathological symptoms, such as anxiety and depression (Nolen-Hoeksema 2000). Ruminating is usually treated as a dysfunctional cognitive coping strategy, as a form of cognitive avoidance; therefore, it may hinder the process of adaptation to trauma and prolong the symptoms of PTSD (Ehring and Ehlers 2014).

Many studies identify a positive relationship between traumatic ruminations and PTSD symptoms (Ehlers and Clark 2000; Ehring and Ehlers 2014; Michael et al. 2007). However, the relationship between rumination and PTSD is complicated by fact that both intrusive and deliberate forms of rumination exist. The former are uncontrolled, destructive, undesirable and automatic thoughts not related to solving problems, while the latter are constructive, more controlled thoughts focused on trying to understand the negative situation and solve the problem (Calhoun et al. 2010; Cann et al., 2011).

Empirical evidence indicates that predominantly ruminative thinking plays a dominant role in the development and maintenance of PTSD (Cann et al. 2011; Ehring and Ehlers 2014; Ehlers and Clark 2000). This has been confirmed by previous studies carried out in Poland among medical rescue workers (Ogińska-Bulik and Juczyński 2016) and in a group of people who experienced various types of trauma, including work-related trauma (Ogińska-Bulik 2016, 2017).

Several studies indicate that a relationship exists between rumination and burnout. One, conducted on a group of healthcare professionals (Vandevala et al. 2017), revealed that affective ruminating was positively related to job burnout and that it acted as a mediator in the relationship between job stressors and burnout, depression and the risk of mental illness. Boren (2014) report a positive relationship between co-ruminations^1^ and burnout in a group of working adults, where co-ruminations also suppressed the relationship between social support and burnout and perceived stress. It should be noted, however, that in Boren (2014), burnout was treated as a dependent variable and ruminations did not refer to an experienced traumatic event.

Burnout, related to negative emotions, may increase the risk of development of PTSD. Its symptoms may also reinforce the cognitive activity that perpetuates the negative aftermath of traumatic events in the form of trauma-related, or intrusive, rumination. This kind of rumination is treated as a dysfunctional cognitive coping strategy, as a form of cognitive avoidance (Ehring and Ehlers 2014), and as a dysfunctional method of emotional regulation (Nolen-Hoeksema et al. 2008). It indicates that trauma-related rumination may mediate the relationship between burnout and PTSD symptoms.

The aim of the study was to determine the mediating role of ruminations in the relationship between burnout and PTSD among police officers exposed to traumatic events related to their work. It was assumed that (1) burnout is positively associated with PTSD symptoms and ruminations, (2) ruminating about experienced traumatic events is positively related to PTSD, and (3) ruminating acts as a mediator in the relationship between burnout and PTSD.

## Methods

Data were obtained from a sample of 120 police officers, representing various departments of the Provincial Police Headquarters and the City Police Headquarters. Of these, 100 (83.3%) confirmed experiencing traumatic events related to work, and only these police officers were included in the analyses. Most of the respondents were male (83%). Their ages ranged from 23 to 47 years (*M* = 33.06, SD = 5.61), and had worked in the police force from 3 to 25 years (*M* = 8.18, SD = 6.07). The survey was voluntary and anonymous. The data were collected with the consent of the superiors during conducted workshops and seminars. The characteristics of the study group are shown in Table 1.

**Table 1:** Characteristics of the examined group

	*N*	%
Sex		
Men	83	83.0
Women	17	17.0
Age		
< 30 years	31	31.0
30–40	57	57.0
> 40	12	12.0
Work experience in police		
< 10 years	63	63.0
10–20	32	32.0
> 20	5	5.0
A police unit		
Antiterrorists	27	27.0
Prevention	43	43.0
Road traffic	30	30.0

Participants provided basic information on age, gender, seniority at work, and completed three standard measurement tools, described briefly below.

The *Posttraumatic Checklist for DSM-5–PCL-5,* authors: Weathers et al. (2013), in the Polish adaptation by Ogińska-Bulik et al. (2018), is used to assess PTSD symptoms. It contains 20 items representing four subscales: re-experience (intrusion), avoidance, negative changes in cognition or mood, and increase in arousal or reactivity. The answers are marked on a 5-point scale, where 0 means *not at all* and 4 means *very strong*. The tool is characterized by very good psychometric properties, including a Cronbach's alpha of 0.96 for the overall PCL-5 result.

The *Oldenburg Burnout Inventory–OLBI*, developed by Halbelsleben and Demerouti (2005), is a 16-element questionnaire used to assess the level of burnout and its two components: exhaustion and disengagement. The answers are given on a scale from 1 (*strongly agree*) to 5 (*strongly disagree*). All responses have been coded so that high scores refer to a high level of exhaustion and lack of commitment. The study used the Polish version of the inventory developed by Cieślak. Both the exhaustion (Cronbach’s *α* = 0.86) and disengagement (0.80) scales are reliable.

The *Event-Related Rumination Inventory–ERRI*, developed by Cann et al. (2011) is a questionnaire used to assess the severity of ruminations associated with an experienced negative life event. The Polish adaptation was developed by Ogińska-Bulik and Juczyński (2015). The inventory contains two scales, each of which consists of 10 statements: the first scale relates to intrusive ruminations, the second to deliberate ruminations. The respondent uses a four-point Likert scale (0—*not at all* to 3—*often*) to evaluate the frequency of occurrence of the situation described in the inventory over a period of several weeks of sustained events. The results are calculated separately for both scales. The two scales demonstrate high internal consistency, with Cronbach's alpha coefficients of 0.96 for the intrusive ruminations scale and 0.92 for the deliberate ruminations scale.

## Results

### Descriptive characteristics of the examined variables

The examined police officers reported experiencing various types of traumatic events related to their work. The most frequently encountered were events directly threatening the life or personal health (71%), events threatening the life or health of a colleague/co-workers (68%), events in which victims died (63%), were attacked (54%) and events in which children were harmed (50%). Other traumatic events included participation in an accident (43%), witnessing a frightening scene (39%) and various others (25%).

Statistical calculations were performed using Statistica 13.1. The mean values of the analyzed variables, PTSD symptoms, burnout and ruminations are presented in Table 2. First, the normality of the distribution of the results was checked. In the case of PTSD and intrusive ruminations, the distributions deviate from normal; therefore, the values were log-transformed to improve the skewness and kurtosis indicators before analysis.

**Table 2:** Descriptive statistics of analyzed variables (*N* = 100)

	*M*	SD	Skewness	Kurtosis
PTSD–total score	0.66	0.69	1.61	2.52
Re-experience	0.66	0.80	1.77	3.16
Avoidance	0.73	0.94	1.42	1.49
Negative alterations in cognitions and mood	0.55	0.66	1.96	4.81
Alterations in arousal and reactivity	0.77	0.80	1.41	1.99
Job burnout–total score	2.66	0.64	-0.16	0.66
Exhaustion	2.58	0.70	0.16	0.47
Disengagement	2.74	0.68	-0.27	0.50
Intrusive ruminations	0.59	0.65	1.75	3.56
Deliberate ruminations	0.80	0.64	0.88	0.74

The examined police officers generally showed a low level of PTSD symptoms, with only 10% of the total number of respondents demonstrating high severity according to the criteria adopted for PCL-5 (cut-off point > 33 points; Ogińska-Bulik et al. 2018), indicating a high probability of occurrence of PTSD. In turn, 39% of the respondents revealed a high level of both exhaustion and distance from work. The surveyed police officers also displayed a similar intensity of burnout syndrome as obtained in a study of healthcare professionals (Shoji et al. 2015); however, in contrast to this group, the surveyed police officers were characterized by a greater severity of withdrawal than exhaustion, albeit an insignificant one.

The police officers demonstrated a relatively low intensity of both types of rumination (*M* = 4 sten, i.e., the upper limit of low results). Based on standardization data by Ogińska-Bulik and Juczyński (2015), 6% of respondents demonstrated a high intensity of intrusive ruminations, and 9% a high intensity of intentional ruminations. Neither gender, age, professional experience nor the represented police unit was significantly related to the analyzed variables. The results were not analyzed separately with regard to the type of injury, because 85% of the surveyed police had suffered an injury directly and 94% indirectly.

### Ruminations as a mediator

Table 3 shows the relationships between the studied variables, i.e., the degree of occupational burnout, symptoms of PTSD, and ruminations about experienced events. This relationship was evaluated based on Pearson’s correlation coefficient.

**Table 3:** Correlation coefficients between burnout, PTSD symptoms and ruminations

	Variables	1	2	3	4	5	6	7	8	9	10
1	Burnout–total	–									
2	Exhaustion	0.93***	–								
3	Disengagement	0.93***	0.72***	–							
4	PTSD–total score	0.38***	0.38***	0.33***	–						
5	Re-experience	0.30**	0.30**	0.25*	0.88***	–					
6	Avoidance	0.27**	0.27**	0.24*	0.85***	0.74***	–				
7	Negative alterations in cognitions and mood	0.37***	0.39***	0.30**	0.92***	0.72***	0.78***	–			
8	Alterations in arousal and reactivity	0.38***	0.36***	0.35***	0.91***	0.71***	0.68***	0.77***	–		
9	Intrusive ruminations	0.37***	0.37***	0.32***	0.80***	0.69***	0.69***	0.72***	0.74***	–	
10	Deliberate ruminations	0.21*	0.25*	0.15	0.64***	0.52***	0.62***	0.64***	0.54***	0.66***	–

*p < 0.01 **p < 0.01 ***p < 0.001

The data presented in the table indicate a positive association between burnout and both PTSD symptoms and ruminations of experienced traumatic situations, especially those of an intrusive nature. Slightly higher correlation coefficients were observed for exhaustion. In addition, strong positive links were found between rumination and PTSD symptoms, with higher values of correlation coefficients associated with intrusive rumination.

The obtained results formed the basis for identifying more complex relationships between variables. In the next stage of the analysis, it was checked whether ruminations act as mediators in the relationship between burnout and PTSD. Mediation analysis was performed using 5000 bootstrap samples according to the bootstrapping procedure proposed by Preacher and Hayes (2008). This method has a high explanatory power, does not require a normal distribution of variables, and allows analyses to be conducted on relatively small samples.

Mediation analysis allows a more complex structure of the model to be analyzed, thus determining whether the variable acting as a predictor (in this case, burnout dimensions) is related to the dependent variable (PTSD) via a third variable (two types of ruminations) that serves as a mediator. The mediating effect occurs when an intermediary variable decreases the predictive value of the independent variable for the dependent variable.

**Fig. 1:**
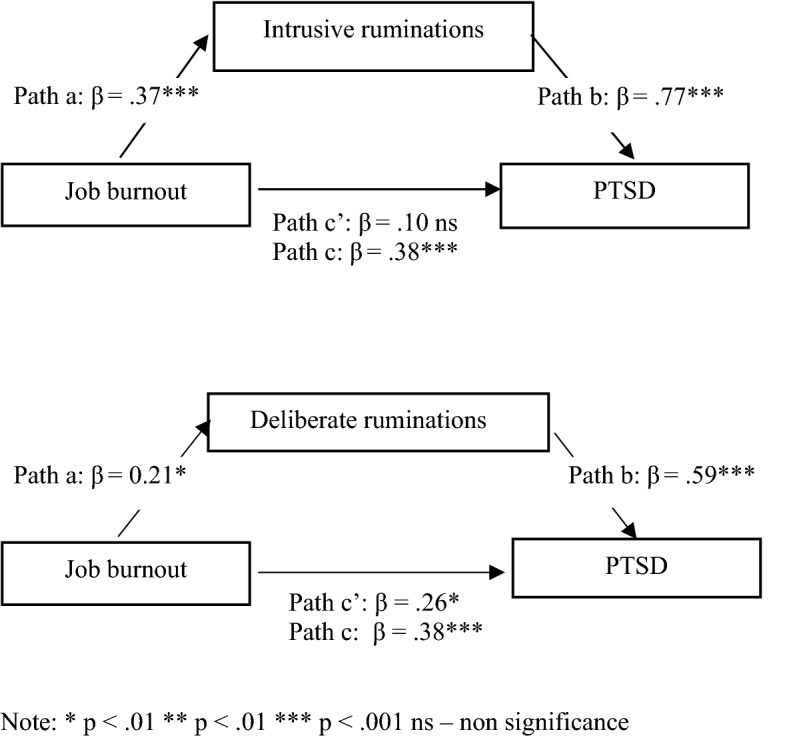
Models of relation between job burnout, ruminations and PTSD symptoms

**Fig. 2:**
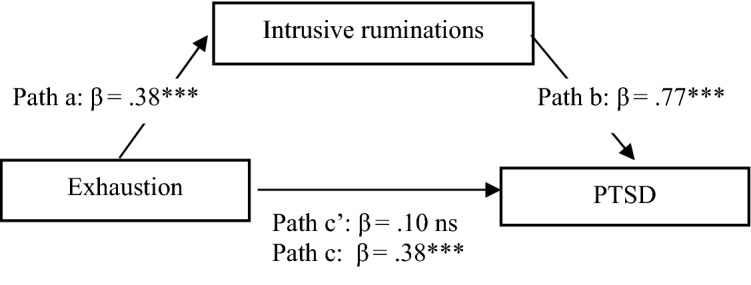
Models of relation between exhaustion, ruminations and PTSD symptoms

**Fig. 3:**
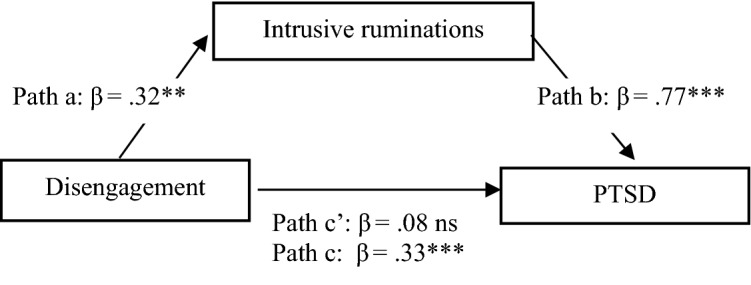
Models of relation between disengagement, ruminations and PTSD symptoms

In total, three models of mediation were obtained in the study, presented in Figs. 1, 2, and 3. *βc* indicates the predictive value of an independent variable before implementing the mediator and *βc*, that of an independent variable after implementing the mediator.

Figure 1 represents job burnout as a predictor of intrusive and deliberative ruminations and PTSD. Both types of ruminations also predict PTSD. The relationships are positive, which indicates that higher levels of job burnout are associated with higher the intensities of intrusive rumination and PTSD. In addition, higher levels of rumination indicate a higher intensity of PTSD. The introduction of intrusive ruminating as an intermediate variable in the relationship between job burnout and PTSD caused this relation to become non-significant, which indicates full mediation. Intrusive ruminating becomes the only predictor of PTSD. Introduction of deliberate rumination as a mediator in the relationship between burnout and PTSD weakens it, which indicates partial mediation.

In the next steps, two separate dimensions of burnout were analyzed as independent variables. Intrusive ruminating fully mediates the relationships between exhaustion and PTSD (Fig. 2) and between disengagement and PTSD (Fig. 3), confirming the significance of this kind of rumination.

## Discussion

The most frequently experienced traumatic situations at work reported by the investigated police officers were events directly threatening their own health or life. However, the reported intensity of PTSD symptoms is low, as opposed to the severity of burnout symptoms. Intrusive ruminating fully mediates between burnout and PTSD, as indicated in model 1, while deliberate ruminating partly mediates this relationship. Considering the two separate burnout dimensions as independent variables, it was confirmed that intrusive rumination fully mediates the relationships between exhaustion and PTSD and between disengagement and PTSD (models 2 and 3), indicating the importance of this type of rumination. Burnout is related to PTSD symptoms both directly and indirectly through rumination about the experienced events. It indicates that police officers who are experiencing burnout and who additionally tend to ruminate about experienced traumatic events are more likely to develop PTSD than those who are only experiencing burnout.

The confirmation that burnout has a predictive role for PTSD indicates that officers experiencing burnout have less energy to deal with traumatic stressors, and as a result, are more prone to PTSD. Our present findings confirm those presented in other studies (Jo et al. 2018; Katsavouni et al. 2016; LaFauci Schutt and Marotta 2011; Maslach and Leiter 2016; Mealer et al. 2009; Wapperom 2016). The findings are in line with the assumptions of *Hobfoll's COR*, i.e., that resources obtained by employees are exhausted by expenditure on work-related stressors. This enhances the negative effects of occupational stress in the form of burnout and makes employees more susceptible to PTSD when exposed to traumatic stress.

Available studies also indicate that job burnout is associated with a decrease in personal and social resources (Ito and Brotheridge 2003). In turn, Shoji et al. (2015) emphasize that the spirals of loss caused by high levels of burnout and limited resources remain a critical factor when dealing with indirect exposure to traumatic events. Therefore, it is desirable to protect existing resources and develop new ones. This can be done by participating in prevention and treatment programs aimed at developing both personal (i.e., self-efficacy, resilience) and environmental (social support) resources. It is also desirable to improve the ability to cope with stress, which can limit the development of burnout. Shoji et al. (2015) indicate the importance of including other resources, such as control beliefs or self-efficacy, in resource prevention programs. A later meta-analysis showed that such beliefs about one’s own ability to cope with stress and its consequences can reduce the symptoms of burnout (Shoji et al. 2016).

Burnout and PTSD are associated with symptoms of apathy, fatigue, cynicism, sleep disorders, generalized irritability and a lack of interest in seeking help, and both may result from low levels of social support. On the other hand, burnout is treated as a response to chronic stressors at work, while PTSD is a response to high-intensity sudden stress that does not have to be associated with professional performance (Bakker and Costa 2014).

It is noteworthy that rumination seems to play a more important role in the development and maintenance of PTSD than burnout. This observation is consistent with trauma cognitive theories, indicating that individual cognitive activity in the face of trauma, and especially ruminating, is one of the most important factors affecting the negative consequences of experienced events (Ehlers and Clark 2000). Ruminating seems to be the mechanism explaining the relationship between burnout and PTSD.

It should be noted that although intrusive ruminations mainly favor the occurrence of PTSD, they may also foster positive changes, expressed in the form of posttraumatic growth (Ogińska-Bulik 2016). Intrusive thinking, which is a natural reaction after experiencing a traumatic event, can be a prelude to conscious rumination, acting to find ways of coping with this situation. In other words, intrusive ruminations give survivors a chance for further cognitive processing of a traumatic event, which may result in posttraumatic growth (Calhoun et al. 2010).

Our research has some limitations. The measurement of PTSD, burnout and rumination was made on the basis of self-assessment. The study was conducted on a relatively small group of police officers, mostly men. It was cross-sectional study, which is an economical and efficient method of testing research hypotheses. Attention should also be paid to the possible overlap between intrusive ruminations and PTSD intrusion. Therefore, the obtained results should be treated with caution and conclusions about the cause–effect relationship should be drawn cautiously. This is particularly important because inverse relationships are possible between variables. A cross-sectional study of traumatic life events, PTSD and ADHD by Brattberg (2006) based on multiple regression analysis found PTSD to be a predictor of occupational burnout; however, the significance of events experienced directly and indirectly was not taken into account. This indirect exposure may lead to negative effects in workers, referred to as *secondary traumatic stress* effects.

Despite its aforementioned limitations, the research nevertheless enriches knowledge about the negative impact of occupational stress on employees of emergency services. It also increases the understanding the relationship between burnout and PTSD among police officers and the indirect role of rumination. It is the first study to identify a relationship between burnout and PTSD in a group of police officers, a group of professionals frequently exposed to both chronic and traumatic stressors that can lead to negative consequences in the form of burnout and PTSD symptoms. Although our findings indicate that job burnout is associated with PTSD, it is primarily its disengagement component that has a stronger relationship with PTSD. These findings contradict those of Brattberg (2006) and Wapperom (2016), who report an overlap between exhaustion and PTSD. In addition, another strength of the present study is that it uses standardized, reliable and relevant research tools.

The Police service is subject to high costs associated with the treatment, absenteeism or employee turnover incurred due to stress and burnout. It is important that police officers who meet the burnout criteria are monitored, as our findings show that they are more likely to experience PTSD symptoms. Future studies should build on our present findings by including other sources of information about the consequences of experienced stress among police officers, such as job interviews. It is also worth considering the role of social support and coping strategies in controlling burnout. In addition, to determine whether burnout in police officers precedes PTSD symptoms, new study designs should be applied, such as longitudinal studies.

Predicting burnout symptoms and posttraumatic stress symptoms allows preventive action to be taken. Such measures are especially important, as burnout is typically a process that develops over a number of years, and early recognition of its development is a crucial step in reducing the likelihood of other negative consequences associated with occupational stress.

The negative effects of occupational stress can be weakened and mitigated by various interventions, such as immediate debriefing and reflection on traumatic effects (Hooper et al. 2010). Pre-implementation programs, as well as training and awareness programs, should be offered to familiarize staff with symptoms of occupational stress such as burnout and PTSD. These intervention programs should include training on positive coping strategies that can be used in stressful and traumatic work situations. Intrusive rumination, as a rule, increases the feeling of anxiety and severity of PTSD symptoms, while deliberate rumination is directed more towards seeking ways of dealing with the experienced traumatic events. In addition, in connection with the introduction of burnout diagnosis, healthcare should offer counseling programs not only about the effects of traumatic events, but also burnout.

It seems appropriate to create a portal and a range of training courses for police officers. The approach should be a gradual one, i.e., progressing from (1) raising awareness of the importance of managing work-related stress; (2) conducting screening to identify determinants of stress and burnout at work; (3) setting intervention goals and selecting appropriate preventive interventions; (4) implementing selected stress management interventions in the workplace; (5) assessing the impact of strategies on work-related stress and burnout. Such portals have already been implemented, for example, the Netherlands Stress-Prevention @ Work for healthcare professionals (Wijnen et al. 2020).

However, a review of research on the effectiveness of interventions related to stress management in police officers, developed by Patterson et al. (2012), indicates that they have practically no significant impact on psychological, behavioral or physiological factors experienced by the recipients. Some approaches focus on organizational factors while others examine individual factors. It is recommended that stress management interventions for police officers should focus on specific types of stress (i.e., organizational or personal); however, more rigorous research is needed to assess the effectiveness of such interventions. A more thorough understanding of the potential for police officers to experience negative psychological effects of a stressful and demanding profession will allow more targeted assistance and improve the overall effectiveness of the profession.

## Data Availability

The datasets used and/or analysed during the current study are available from the corresponding author on reasonable request.

## Footnotes

1 Co-rumination means excessively discussing personal problems within a dyadic relationship and is related to the psychological concept of rumination.

## References

[CR1] Aamodt M, Stalnaker N (2006) Police officer suicide: frequency and officer profiles. https://www.police1.com/health-fitness/articles/police-officer-suicide-frequency-and-officer-profiles-HFJ5hMgo5cnq6fA0/

[CR2] American Psychiatric Association (2013). DSM-5.

[CR3] Aydin R, Tekiner MA (2016). Analysis of burnout level of police officers: evidence from Malatya, Turkey. J Trans Stud.

[CR4] Backteman-Erlanson S, Padyab M, Brulin C (2013). Prevalence of burnout and associations with psychosocial work environment, physical strain, and stress of conscience among Swedish female and male police personnel. Police Pract Res.

[CR5] Bakker AB, Costa PL (2014). Chronic job burnout and daily functioning: a theoretical analysis. Burn Res.

[CR6] Basinska B, Daderman AM (2014). Fatigue and burnout in police officers: The mediating role of emotions. POLICING.

[CR7] Basinska B, Gruszczyńska E (2017). Positivity and job burnout in emergency personnel: examining linear and curvilinear relationship. Pol Psychol Bull.

[CR8] Boren JP (2014). The relationships between co-rumination, social support, stress, and burnout among working adults. Manag Commun Q.

[CR9] Brattberg G (2006). PTSD and ADHD: Underlying factors in many cases of burnout. Stress Health.

[CR10] Calhoun LG, Cann A, Tedeschi RG, Weiss T, Berger R (2010). The posttraumatic growth model: Sociocultural considerations. Posttraumatic growth and culturally competent practice: Lessons learned from around the globe.

[CR11] Cann A, Calhoun LG, Tedeschi RG, Triplett KN, Vishnevsky T, Lindstrom CM (2011). Assessing posttraumatic cognitive processes: The Event Related Rumination Inventory. Anx Stress Coping.

[CR12] De la Fuente Solana E, Aguayo RE, Vargas CP, de la Fuente C (2013). Prevalence and risk factors of burnout syndrome among Spanish police officers. Psicothema.

[CR13] Demerouti E, Bakker AB, Vardakou I, Kantas A (2003). The convergent validity of two burnout instruments. Eur J Psychol Assess.

[CR14] Demerouti E, Mostert K, Bakker AB (2010). Burnout and work engagement: a thorough investigation of the independency of both constructs. J Occup Health Psychol.

[CR15] Dudek B (2003). Zaburzenie po stresie traumatycznym. [Post-traumatic Stress Disorder].

[CR16] Ehlers A, Clark DM (2000). A cognitive model of posttraumatic stress disorder. Behav Res Ther.

[CR17] Ehring T, Ehlers A (2014). Does rumination mediate the relationship between emotion regulation ability and posttraumatic stress disorder?. Eur J Psychotraumatol.

[CR18] Foa EB, Rothbaum BO (1998). Treating the trauma of rape: cognitive-behavioral therapy for PTSD.

[CR20] Halbesleben JR, Demerouti E (2005). The construct validity of an alternative measure of burnout: investigating the English translation of the Oldenburg Burnout Inventory. Work Stress.

[CR21] Hartley TA, Violanti JM, Sarkisian K, Andrew ME, Burchfiel CM (2013). PTSD symptoms among police officers: associations with frequency, recency, and types of traumatic events. Int J Emerg Ment Health.

[CR22] Hobfoll SE, Freedy J, Schaufeli WB, Maslach C, Marek T (1993). Conservation of resources: a general stress theory applied to burnout. Professional burnout: recent developments in theory and research.

[CR23] Hooper C, Craig J, Janvrin DR, Wetzel MA, Reimels E (2010). Compassion satisfaction, burnout and compassion fatigue among emergency nurses compared with nurses in other selected inpatient specialties. J Emerg Nurs.

[CR24] Horowitz MJ (1986). Stress response syndrome.

[CR25] Ito JK, Brotheridge CM (2003). Resources, coping strategies, and emotional exhaustion: a conservation of resources perspective. J Vocat Behav.

[CR26] Janoff-Bulman R (1992). Shattered assumptions. Towards a new psychology of trauma.

[CR27] Jo I, Lee S, Sung G, Kim M, Lee S, Park J, Lee K (2018). Relationship between burnout and PTSD symptoms in firefighters: the moderating effects of a sense of calling to firefighting. Int Arch Occup Environ Health.

[CR28] Katsavouni F, Bebetsos E, Malliou P, Beneka A (2016). The relationship between burnout, PTSD symptoms and injuries in firefighters. Occup Med.

[CR29] LaFauci Schutt JM, Marotta SA (2011). Personal and environmental predictors of posttraumatic stress in emergency management professionals. Psychol Trauma.

[CR30] Marchand A, Boyer R, Nadeau C, Martin M (2015). Predictors of posttraumatic stress disorders in police officers: a prospective study. Psychol Trauma.

[CR31] Maslach C, Leiter M (2016). Understanding the burnout experience: recent research and its implications for psychiatry. World Psychiatry.

[CR32] Maslach C, Schaufeli WB, Leiter MP (2001). Job burnout. Ann Rev Psychol.

[CR33] Mealer M, Burnham EL, Goode CJ, Rothbaum B, Moss M (2009). The prevalence and impact of post-traumatic stress disorder and burnout syndrome in nurses. Depress Anxiety.

[CR34] Michael T, Halligan SL, Clark DM, Ehlers A (2007). Rumination in posttraumatic stress disorder. Depress Anxiety.

[CR35] Nolen-Hoeksema S (2000). The role of rumination in depressive disorders and mixed depressive/anxiety symptoms. J Abnorm Psychol.

[CR36] Nolen-Hoeksema S, Wisco BE, Lyubomirsky S (2008). Rethinking rumination. Perspect Psychol Sci.

[CR37] Ogińska-Bulik N (2016). The role of rumination in the occurrence of positive effects of experienced traumatic events. Health Psychol Rep.

[CR38] Ogińska-Bulik N (2017). Negative and positive effects of the exposure to trauma among soldiers participating in military missions: the role of rumination. Adv Psychiatry Neurol.

[CR39] Ogińska-Bulik N, Juczyński Z (2015). The Polish adaptation of the event related rumination inventory. Rev Psychol.

[CR40] Ogińska-Bulik N, Juczyński Z (2016). Ruminations as predictors of negative and positive effects of experienced traumatic event in medical rescue workers. Occup Med.

[CR41] Ogińska-Bulik N, Juczyński Z, Lis-Turlejska M, Merecz-Kot D (2018). Polish adaptation of PTSD Checklist for DSM-5–PCL-5. A preliminary communication. Rev Psychol.

[CR43] Patterson GP, Chung IW, Swan PW (2012). Stress management interventions for police officers and recruits: a meta-analysis. J Exp Criminol.

[CR44] Preacher K, Hayes AF (2008). Asymptotic and resampling strategies for assessing and comparing indirect effects in multiple mediator models. Behav Res Methods.

[CR45] Shoji K, Lesnierowska M, Smoktunowicz E, Bock J, Luszczynska A, Benight Ch, Cieślak R (2015). What comes first, job burnout or secondary traumatic stress? Findings from two longitudinal studies from the US and Poland. PLoS ONE.

[CR46] Shoji K, Cieślak R, Smoktunowicz E, Rogala A, Benight CH, Łuszczyńska A (2016). Association between job burnout and self-efficacy: a meta-analysis. Anxiety Stress Coping.

[CR47] Vandevala T, Pavey L, Chelidoni O, Chang NF, Creagh-Brown B, Cox A (2017). Psychological rumination and recovery from work in intensive care professionals: associations with stress, burnout, depression and health. J Intensive Care.

[CR48] Wapperom A (2016) Burnout: a risk factor for developing PTSD? Master thesis. Utrecht University, The Netherlands

[CR49] Weathers F, Litz B, Keane T, Palmieri P, Marx B, Schnurr P (2013) The PTSD Checklist for DSM–5 (PCL-5). Scale available from the National Center for PTSD. https://www.ptsd.va.gov/professional/assessment/adult-sr/ptsd-checklist.asp

[CR50] Wijnen BF, Lokkerbol J, Boot C, Havermans BM, van der Beek AJ (2020). Smit F (2019) Implementing interventions to reduce work-related stress among health-care workers: an investment appraisal from the employer’s perspective. Int Arch Occup Environ Health.

